# A New Sphygmoscope

**Published:** 1880-10

**Authors:** 


A SPHYGMOSCOPE Has been made by Dr. F. P. Stevens, of New York City, that promises much usefulness in the investigation of certain conditions of the circulatory apparatus.

Its cheapness, simplicity and readiness of application, are all strong recommendations in its favor, and will doubtless contribute largely towards its general adoption. The Sphygmograph on the contrary, is an expensive and complex instrument, and is far from being entitled to all the eulogiums which a few have been pleased to lavish upon it. In fact, it would not be difficult to show, that some of the advantages which have been claimed for it, can be *reached* by nothing short of the *educated human touch*, under the control of an intelligent brain, and that no mechanical contrivance can ever be constructed that will equal this in diagnosis.

It is a complicated little apparatus, useful in some cases in aiding one to *see* certain states or conditions of the circulation on paper or other substance, and thus by a co-relation of the senses of sight and touch, develope points, which neither sense, without mechanical assistance, would be able to establish. Beyond this aid to an educated and delicate touch, and the *impression* it may have upon the patient or lookers on, it is really an unimportant little machine, except as a rather expensive toy to the owner.

